# Slamming sex and psychotic symptoms. A case report

**DOI:** 10.1192/j.eurpsy.2021.1711

**Published:** 2021-08-13

**Authors:** A. Sotillos Gómez, A. Hernández Mata, P. Marco Coscujuela

**Affiliations:** Psychiatry, Hospital Universitario de Getafe, Getafe, Spain

**Keywords:** psychotic symptoms, chemsex, slamsex, drugs-induced psycosis

## Abstract

**Introduction:**

Chemsex is the term used to describe the use of psichoactives drugs to practice sex, mostly among men who have sex with other men. When drugs are administered by intravenously it is know as slamming or slamsex. Mephedrone is drug more used to this practice, in combination with other as anfetamines. This practice has been associated with a lot of psychiatric and organic complications.

**Objectives:**

Describe a case about one of chemsex complications such as drug- induce psychosis. Moreover, show the multiple medical complications associated with this practice.

**Methods:**

Patient’s data is obtained from medical history, psychiatric interviews carried out during his hospitalizations and his psychological follow-up in CAID.

**Results:**

45 year-old man patient was admitted into a psychiatric unit due to paranoid ideation, behavioral disturbances and heteroaggressive behavior after mephedrone, amphetamines and other drugs intoxication in the context of slamsex practice. He has a history of two previous autolytic attempts but no psychotic episodes. After one week of hospitalization and antipsychotic treatment psychotic symptons disappear. Concerning his medical history, he was infected for HIV, syphilis, hepatitis A, visceral Leishmania.

**Conclusions:**

It is necessary to be aware of the increased in chemsex and slamsex rates and therefore of the comorbilities that have associated. Rapid detection is important in order to reduce and control the severe addiction they entail (especially intravenous consumption).

**Disclosure:**

No significant relationships.

